# Semen analysis in fertile patients undergoing vasectomy: reference values and variations according to age, length of sexual abstinence, seasonality, smoking habits and caffeine intake

**DOI:** 10.1590/S1516-31802005000400002

**Published:** 2005-07-07

**Authors:** Bernardo Passos Sobreiro, Antonio Marmo Lucon, Fábio Firmbach Pasqualotto, Jorge Hallak, Kelly Silveira Athayde, Sami Arap

**Keywords:** Semen, Age groups, Sexual abstinence, Smoking, Caffeine, Sêmen, Grupos etários, Abstinência sexual, Tabagismo, Cafeína

## Abstract

**CONTEXT AND OBJECTIVE:**

Recent studies have shown regional and population differences in semen characteristics. The objective was to establish reference values for semen analysis and to verify the effect that age, length of sexual abstinence, seasonality, smoking habits and coffee consumption have on fertile individuals' semen characteristics.

**DESIGN AND SETTING:**

Prospective study in the Urology Division, Hospital das Clínicas, Universidade de São Paulo.

**METHODS:**

Between September 1999 and August 2002, 500 fertile men requesting a vasectomy for sterilization purposes were asked to provide a semen sample before the vasectomy. We evaluated the effects of age, sexual abstinence, seasonality, smoking and coffee consumption on semen characteristics.

**RESULTS:**

Compared with World Health Organization values, 87.2% of the patients presented sperm morphology below the normal level. A significant decline in semen volume, sperm motility and sperm morphology in patients over 45 years of age was observed. In patients with 5 days or more of abstinence, there was reduced sperm motility. The lowest values for sperm concentration, motility and morphology were observed in summer and the highest in winter. No differences in semen parameters relating to smoking were detected. Patients who drank six or more cups of coffee per day presented higher sperm motility.

**CONCLUSIONS:**

Our sample had a very low percentage of normal sperm morphology. Only sperm morphology showed a high abnormality rate. Differences in semen parameters with regard to age, length of sexual abstinence, seasonality and coffee consumption were identified. No differences relating to smoking were detected.

## RESUMO

**CONTEXTO E OBJETIVO:**

Estudos recentes têm demonstrado diferenças regionais e populacionais quanto a características do sêmen. O objetivo foi estabelecer valores de referência para análise seminal e verificar o efeito da idade, tempo de abstinência sexual, sazonalidade, hábito de fumar e consumo de cafeína sobre as características do sêmen de indivíduos férteis.

**TIPO DE ESTUDO E LOCAL:**

Estudo prospectivo, na Divisão de Clínica Urológica do Hospital das Clínicas, Universidade de São Paulo.

**MÉTODOS:**

Entre setembro de 1999 e agosto de 2002, 500 homens férteis requisitando vasectomia com propósitos de esterilização voluntária foram solicitados a coletar uma amostra de sêmen antes da vasectomia. Avaliamos efeitos da idade, abstinência sexual, sazonalidade, consumo de cigarros e café nas características seminais.

**RESULTADOS:**

Quando comparados aos valores da Organização Mundial de Saúde, 87,2% dos pacientes apresentavam morfologia espermática abaixo do normal. Foi observado declínio significativo do volume seminal, motilidade e morfologia em pacientes acima de 45 anos. Em pacientes com cinco dias ou mais de abstinência, houve redução da motilidade. Valores mais baixos para concentração, motilidade e morfologia espermática foram observados no verão e os mais elevados no inverno. Não houve diferenças nos parâmetros seminais estudados de acordo com o hábito de fumar. Com relação ao consumo de café, pacientes que ingeriam seis ou mais xícaras/dia apresentaram maior motilidade espermática.

**CONCLUSÃO:**

Houve baixa porcentagem de morfologia espermática normal. Apenas a morfologia espermática apresentou alto percentual de anormalidade se levado em consideração o padrão descrito pela Organização Mundial de Saúde. Foram identificadas diferenças nos parâmetros seminais de acordo com a idade, tempo de abstinência sexual, sazonalidade e consumo de café. Não houve diferenças de acordo com o hábito de fumar.

## INTRODUCTION

In view of the need to standardize laboratory techniques and determine normal semen parameters, the World Heath Organization (WHO) published its *Laboratory Manual for Examination of Human Semen and Semen-Cervical Mucus Interaction*, in 1980.^1^ Subsequently, revisions were published in 1987,^2^ 1992^3^ and 1999.^4^ However, due to the differences between populations, the WHO itself recommends that each laboratory should establish its own reference values (WHO, 1999).^4^

The importance of each laboratory determining its own values lies in the fact that fertile individuals may occasionally be considered subfertile or even incapable of establishing pregnancy when the semen analysis is compared with values established for other populations.^5^ This may give rise to unnecessary treatment and embarrassing situations for couples in which the woman has a spontaneous pregnancy by a man who was considered subfertile according to the WHO parameters.^6,7^ Furthermore, for assisted reproduction techniques, and especially intracytoplasmic sperm injection (ICSI) into the oocyte, semen analysis should address the factors responsible for failure of such techniques. The traditional parameters are thus merely descriptive.^8^

Various factors may be associated with variations in sperm characteristics among fertile individuals. These include increasing age, which is associated with diminution of semen volume, sperm motility and sperm morphology.^9,10^ A meta-analysis study has demonstrated that smokers present an average reduction of 13% in sperm concentration, 10% in sperm motility and 3% in sperm morphology.^11^ Seasonal variations in sperm quality, with greater concentration, motility and morphology in winter than in summer, have also been reported.^12^ The length of sexual abstinence preceding the collection of sperm for analysis is an important variable for the quality of the test. Very short or very long periods of abstinence are associated with distorted semen analysis results (WHO, 1999).^4^ Individuals who drink coffee every day present increased sperm motility, although they present no significant alterations in the concentration or morphology of the sperm.^13^

The present study had the objectives of establishing reference values for semen analysis for a population of fertile individuals in the city of São Paulo, and identifying variations in semen characteristics according to age, length of sexual abstinence, seasonality, smoking habits and coffee consumption.

## METHODS

Five hundred fertile candidates for voluntary sterilization by vasectomy were included in this study project between September 1999 and August 2002. Their average age was 35.04 ± 6.1 years (range: 24 to 63).

In the hospital, a sperm sample was collected in the morning, from each individual by masturbation, after two to five days of sexual abstinence. A wide-necked sterile non-toxic flask was utilized for sample collection. The sample was protected from extremes of temperature (< 20° C or > 40° C) during the laboratory process. Liquefaction was performed, followed immediately by an assessment of semen color, volume (using a graduated pipette), viscosity and pH. Ten microliters of semen were deposited in a Makler sperm-count chamber and the sperm concentration (expressed as 10^6^/ml) was determined by phase microscopy, at 200 x magnification. The sperm motility assessment was carried out by making two counts of 100 spermatozoa. In the event of a discrepancy of more than 10% between the two readings, a third analysis was made and the final result was taken to be the arithmetical mean of the three assessments. Sperm morphology was determined by the semen smear technique, using two smear samples of 10 ml of fresh semen. Panoptic staining was utilized for the smear slides. At least 200 spermatozoa were studied under 1000 x magnification and were classified in accordance with the World Health Organization descriptions (1992).^6^ The reference values for the semen parameters were expressed as arithmetical mean and standard deviation. The 25^th^, 50^th^ and 75^th^ percentiles were determined for each variable.

To study the influence of the different variables on the semen parameters, the patients were divided into groups. For age, the groups were: 24-30 years (n = 117); 31-35 years (n =166); 36-40 years (n = 127); 41-45 years (n =59); and > 45 years (n = 31). For the length of sexual abstinence, the groups were: 2 days (n = 47); 3 days (n = 199); 4 days (n = 101); 5 days (n = 99); and > 5 days (n = 54). For the month of the year, the groups were: January (n = 58); February (n = 20); March (n = 62); April (n = 39); May (n = 38); June (n = 26); July (n = 29); August (n = 41); September (n = 35); October (n = 53); November (n = 52); and December (n = 47). For smoking habit, the groups were: non-smokers (n = 324); 1-10 cigarettes/day (n = 80); 11-20 cigarettes/day (n = 66); and > 20 cigarettes/day (n = 30). For coffee consumption, the groups were: no coffee consumption (n = 151); 1-3 cups of coffee per day (n = 249); 4-6 cups/day (n = 48); and > 6 cups/day (n = 52).

The distribution of the variables was "normalized" by logarithmic transformation. Variance analysis (ANOVA) and Bonferroni's post-hoc test were used for the comparisons between the groups.

The level of statistical significance established was 5%. The data were analyzed by means of the Statistical Package for Social Sciences (SPSS), version 11.0, computer software (SPSS Inc., Chicago, United States).

## RESULTS

The reference values (mean and standard deviation, and 25^th^, 50^th^ and 75^th^ percentiles) for the patients' semen characteristics are presented in Table 1. It can be seen that, in 32% of the cases, the semen volume was below the level considered normal in the WHO descriptions. For other semen parameters, the percentages of patients below WHO levels were 16% for pH, 6% for sperm concentration, 7.8% for total number of spermatozoa, 29.6% for sperm progressive motility and 87.2% for sperm morphology.

**Table 1:** Semen characteristics among 500 fertile individuals and World Health Organization (WHO) reference values

Variable	Average and standard deviation	Percentile			WHO reference values^6^	Proportion of individuals with results below WHO reference values (%)^6^
		25	50	75		
Semen volume (ml)	2.75 ± 1.43	1.6	2.5	3.7	≥ 2.0	32.2
Sperm concentration × 10^6^/ml	99.55 ± 61.11	51.9	87.2	147.4	≥ 20 × 10^6^/ml	6.0
Total number of spermatozoa	283.74 ± 235.41	118.0	225.0	373.0	≥ 40 × 10^6^/ml	7.8
Sperm progressive motility (a + b) (%)	58.65 ± 18.26	47.0	61.0	72.0	≥ 50%	29.6
Normal sperm morphology (%)	17.57 ± 9.50	11.0	16.0	24.0	≥ 30%	87.2

*a = fast; b = slow.*

The variations in semen volume, sperm concentration, sperm progressive motility and sperm morphology in relation to patients' ages are presented in Table 2. This shows that there was a decline in the values of these parameters among individuals over 45 years of age (p < 0.05). There were no statistically significant differences in sperm concentration between the age groups (p < 0.05).

**Table 2:** Mean and standard deviation for semen volume, sperm concentration, sperm motility and normal sperm morphology among 500 fertile individuals, by age group

Age (years)	Semen volume (ml)*	Sperm concentration (10^6^/ml)	Sperm progressive motility (%)	Normal sperm morphology (%)
24 – 30	2.7 ± 1.3^a^	87.4 ± 60.1	60.3 ± 17.3^a^	17.9 ± 9.3^a^
31 – 35	2.9 ± 1.4^a^	98.6 ± 55.2	61.4 ± 14.1^a^	19.0 ± 8.8^a^
36 – 40	2.6 ± 1.5^a^	99.7 ± 61.8	61.9 ± 14.8^a^	18.7 ± 10.0^a^
41 – 45	2.3 ± 1.2^b^	113.8 ± 63.8	56.9 ± 12.5^b^	19.5 ± 9.9^a^
> 45	2.2 ± 1.1^b^	87.8 ± 68.6	53.5 ± 15.8^c^	14.3 ± 9.6^b^
p†	0.0189	0.1189	0.0369	0.0118

* *The values for mean and standard deviation given are presented without logarithmic transformation.*

† *The p-value was established by analysis of variance (ANOVA) and was based on data with logarithmic transformation. Post-hoc Bonferroni test: no differences between groups with the same letter (test does not apply when p ANOVA > 0.05).*

Table 3 shows that the semen volume and sperm concentration increased according to how many days of sexual abstinence there were (p < 0.05). However, in patients with five or more days of sexual abstinence, a reduction in progressive motility (p < 0.05) was found. The sperm morphology did not vary with length of sexual abstinence (p < 0.05).

**Table 3:** Mean and standard deviation for semen volume, sperm concentration, sperm motility and normal sperm morphology among 500 fertile individuals, according to length of sexual abstinence

Number of days of abstinence	Semen volume (%)*	Sperm concentration (10^6^/ml)	Sperm progressive motility (%)	Normal sperm morphology (%)
2	2.3 ± 1.1^a^	105.1 ± 87.7^a^	55.2 ± 16.5^a^	16.4 ± 12.2
3	2.5 ± 1.3^a^	104.8 ± 85.6^a^	61.4 ± 15.3^b^	17.7 ± 9.3
4	2.7 ± 1.3^b^	105.4 ± 61.3^a^	61.6 ± 13.5^b^	18.4 ± 9.2
5	3.0 ± 1.6^c^	127.1 ± 84.7^b^	58.7 ± 15.6^c^	18.0 ± 8.8
>5	3.3 ± 1.7^d^	136.1 ± 82.1^c^	56.3 ± 16.0^d^	17.0 ± 9.1
p*†*	0.011	0.022	0.020	0.186

* *The values for mean and standard deviation given are presented without logarithmic transformation.*

† *The p-value was established by analysis of variance (ANOVA) and was based on data with logarithmic transformation. Post-hoc Bonferroni test: no differences between groups with the same letter (test does not apply when p ANOVA > 0.05).*

From Table 4, it can be seen that the variance analysis demonstrated statistically significant differences for sperm concentration, progressive motility and normal sperm morphology percentage, in relation to the months of the year. When Bonferroni's test was used, statistically significant lower sperm counts were observed in January (85.8 × 10^6^/ml) and February (95.7 × 10^6^/ml), in relation to July (133.4 × 10^6^/ml) and August (129.3 × 10^6^/ml) (p < 0.05). The test did not show any significant differences between the other months of the year. Similarly, it was found that sperm progressive motility was lower in February (54.7%) than in July (67.7%; p < 0.05). The normal sperm morphology percentage was lower in the months of January (15.3%) and February (15.1%) than in July (20.7%; p < 0.05).

**Table 4:** Mean and standard deviation for semen volume, sperm concentration, sperm motility and normal sperm morphology among 500 fertile individuals, by month of the year

Month of the year	Semen volume (%)*	Sperm concentration (10^6^/ml)	Sperm progressive motility (%)	Normal sperm morphology (%)
January	2.9 ± 1.6	85.8 ± 65.7	58.3 ± 13.6	15.3 ± 10.2
February	3.0 ± 1.6	95.7 ± 62.2	54.7 ± 17.1	15.1 ± 8.7
March	2.6 ± 1.4	103.3 ± 60.2	60.2 ± 15.1	18.0 ± 5.4
April	2.5 ± 1.2	125.8 ± 98.0	58.7 ± 18.0	16.7 ± 8.9
May	3.0 ± 1.6	105.7 ± 64.2	55.8 ± 15.8	17.0 ± 7.7
June	3.0 ± 1.7	115.9 ± 60.3	59.8 ± 12.8	16.2 ± 5.8
July	3.4 ± 1.6	133.4 ± 81.5	67.7 ± 14.9	20.7 ± 7.4
August	2.7 ± 1.2	129.3 ± 65.9	64.5 ± 14.4	19.7 ± 8.5
September	2.5 ± 1.4	128.9 ± 66.4	62.4 ± 14.6	170. ± 10.0
October	2.6 ± 1.3	103.6 ± 68.6	59.5 ± 15.8	18.2 ± 12.3
November	2.8 ± 1.5	116.1 ± 81.4	64.1 ± 14.2	19.0 ± 10.5
December	2.3 ± 1.1	116.7 ± 98.2	56.7 ± 16.6	17.0 ± 10.9
p*†*	0.152	0.032	0.003	0.002

* *The values for mean and standard deviation given are presented without logarithmic transformation.*

† *The p-value was established by analysis of variance (ANOVA) and was based on data with logarithmic transformation. Post-hoc Bonferroni test does not apply when p ANOVA > 0.05.*

The semen parameter variations relating to smoking habits are set out in Table 5. No statistically significant differences were observed between smokers and non-smokers, or between the groups of smokers (p < 0.05).

**Table 5:** Mean and standard deviation for semen volume, sperm concentration, sperm motility and normal sperm morphology among 500 fertile individuals, according to smoking habit

Smoking habit (cigarettes/day)	Semen volume (%)*	Sperm concentration (10^6^/ml)	Sperm progressive motility (%)	Normal sperm morphology (%)
0	2.8 ± 1.5	107.9 ± 74.6	59.6 ± 15.3	18.1 ± 9.0
1-10	2.3 ± 1.2	106.6 ± 79.3	58.9 ± 16.7	14.5 ± 7.7
11-20	2.3 ± 1.2	127.9 ± 99.2	61.8 ± 15.8	16.8 ± 10.2
>20	2.1 ± 0.8	130.6 ± 74.9	55.6 ± 13.2	15.7 ± 13.8
p*†*	0.610	0.111	0.529	0.105

* *The values for mean and standard deviation given are presented without logarithmic transformation.*

† *The p-value was established by analysis of variance (ANOVA) and was based on data with logarithmic transformation. Post-hoc Bonferroni test does not apply when p ANOVA > 0.05.*

Table 6 shows that, as coffee consumption increased, so did sperm motility. Among patients who were not in the habit of drinking coffee, progressive motility averaged 57.1%, whereas for the patients who consumed more than six cups of coffee per day, it averaged 62.4% (p < 0.05). There were no significant differences in semen volume, sperm concentration or sperm morphology in relation to coffee consumption (p < 0.05).

**Table 6:** Mean and standard deviation for semen volume, sperm concentration, sperm motility and normal sperm morphology among 500 fertile individuals, according to coffee consumption

Coffee consumption (cups/day)	Semen volume (%)*	Sperm concentration (10^6^/ml)	Sperm progressive motility (%)	Normal sperm morphology (%)
0	2.7 ± 1.5	110.8 ± 79.7	57.1 ± 16.2^a^	17.3 ± 8.2
1 – 3	2.6 ± 1.4	113.6 ± 82.0	60.7 ± 14.6^b^	17.5 ± 10.0
4 – 6	2.7 ± 1.3	111.0 ± 94.8	61.2 ± 15.5^b^	17.9 ± 8.3
>6	2.7 ± 1.7	127.2 ± 8239	62.4 ± 16.0^c^	18.0 ± 9.2
p*†*	0.765	0.634	0.037	0.871

* *The values for mean and standard deviation given are presented without logarithmic transformation.*

† *The p-value was established by analysis of variance (ANOVA) and was based on data with logarithmic transformation. Post-hoc Bonferroni test: no differences between groups with the same letter (test does not apply when p ANOVA > 0.05).*

## DISCUSSION

### Reference standards for sperm analysis

Semen analysis is one of the most important diagnostic methods for the assessment of male infertility. The methodology for semen analysis has been undergoing constant improvement, and new assessment criteria have been proposed.^4^ Similarly, the reference values for each parameter have been the subject of debate. Modifications in sperm quality over the years have been reported and there has apparently been a decline, especially in sperm concentration.^14,15^ Regional differences in sperm parameters have frequently come to light.^16,17^ Thus, it is necessary for each laboratory to establish its own reference values for semen analysis and compare these with the WHO values. In the present study, it was found that only in relation to normal sperm morphology was there a high number of individuals below the WHO standard (87.2% of the individuals studied). For the other variables, the numbers of individuals below the WHO standards ranged from 6% to 32.2%. Further studies will be necessary to determine whether the values obtained are true figures for the sample studied, or whether there are discrepancies in the analysis.

### Age

Various studies using animal models have reported reductions in fertility as age increases. Mice over 18 months of age undergo structural alterations in their germinative cells, with significant reduction in their numbers. Mice aged more than 33 months present an almost total cessation of spermatogenesis.^18^ Testicular atrophy and degeneration of the somniferous epithelium has been observed in rats of advanced age.^19^

In human beings, there is an association between aging and functional decline in the Leydig cells.^20^ Smaller semen volumes and lower sperm motility have been found to accompany advancing age among fertile individuals.^21^ There does not, however, appear to be any reduction in sperm concentration in elderly individuals.^22^ In contrast with the feminine menopause, which is accompanied by a reduction in fertility from the age of 35 years onwards, men may maintain their fertility into advanced old age. One birth of a child to a father aged 70 or over occurs for every 10,000 births of children to fathers aged 30 years.^23^ A large proportion of the individuals who seek assistance in infertility clinics are aged over 65.^24^

The data produced by the present study demonstrate a reduction in semen volume, sperm progressive motility and sperm normal morphology percentage from the age of 45 years onwards, as already described.^21,22^ The sperm concentration was lower in patients over 45 years of age, but this difference was not statistically significant. However, since the majority of the sample for our study consisted of young individuals seeking sterilization by vasectomy, the effect of age on sperm concentration could not be exhaustively assessed.

### Length of sexual abstinence

In fertile individuals, the length of sexual abstinence affects all semen parameters. With abstinence, there is increasing sperm concentration accompanied, however, by decreasing progressive motility and normal morphology percentage.^25^ Similar results have been observed among infertile patients, for whom a prolonged period of sexual abstinence was associated with increased semen volume ejaculated and increased sperm concentration, but with no reduction in progressive motility or normal morphology percentage.^26^ In particular situations, with the use of assisted reproduction techniques for infertile patients, prolonged sexual abstinence may be used for the purpose of obtaining a larger number of spermatozoa. Among 50 individuals with nonobstructive azoospermia who were candidates for testicular biopsy and ICSI into the oocyte, there was an increase in the total number of spermatozoa, although with no change in progressive motility when the period of abstinence was increased from 4 to 14 days.^27^ In the present study, semen volume and sperm concentration increased with the length of abstinence. Sperm progressive motility presented a reduction from the fourth day onwards, and there was no modification of sperm normal morphology percentage with increased length of sexual abstinence.

### Seasonal variation

Seasonal variation in conception rates for human beings has been described, with a reduction in the number of births in the spring as a consequence of a lower conception rate during the summer.^28^ Semen donors have not been found to have any seasonal alteration in sperm motility. However, their sperm concentration has been found to be lower in the summer than in the winter.^29^ Individuals assessed for infertility have presented lower sperm concentration and progressive motility during the summer, with an improvement in these parameters over the winter months.^30^ Lower sperm motility and higher rates of sperm tail defects and immature forms were discovered during the summer months in 2,065 fertile men.^31^ Initially, the variations in semen parameters during the year were attributed to climatic differences. Thus, the high temperatures registered in the summer months were held responsible for the reduction in sperm quality, while it was thought that the low temperatures of the winter months would favor spermatogenesis.^32,33^ However, subsequent studies suggested that temperature was not the only factor involved and that the length of daylight also needed to be considered.^34,35^

Most of the studies have been conducted in countries of the northern hemisphere with large differences in climatic conditions between summer and winter. In Singapore, a tropical country in which the climatic differences are smaller, no differences in semen parameters in relation to the months of the year were found for 7,656 sperm analyses.^36^ The present study, undertaken in the tropical region of Brazil, shows that there are seasonal differences in sperm quality. Factors such as population and climatic differences, and those related to the selection of samples (we studied fertile individuals), make any comparison with the results obtained in Singapore difficult. Generally speaking, the lower sperm concentration, progressive motility and normal morphology percentage found among individuals in São Paulo city during the summer months are similar to those described for populations in the northern hemisphere.

### Smoking

Despite the anti-smoking campaigns carried out throughout the world, the smoking habit is still quite common, affecting 35% of all adult European men^37^ and 33% of the individuals that we studied. It is estimated that, in Brazil, 42% of the population in the southern region smoke: Porto Alegre (capital of the State of Rio Grande do Sul) is the city with the highest figures for lung cancer.^38^ Even though only 31% of the population smoke in the northeastern region of Brazil, this percentage is still considered very high. In our country, it is estimated that approximately 200,000 deaths/year are due to cigarette smoking.^38^

The association between smoking and infertility has already been described, and reductions in sperm concentration, motility and normal morphology percentage among infertile patients who smoke have been ob- served.^39,40^ In a meta-analysis covering 27 studies on sperm quality among smokers, an average reduction of 13% in sperm concentration, 10% in motility and 3% in normal morphology percentage was observed.^11^ The mechanism whereby smoking leads to infertility has still to be clarified. Hormone alterations are present in smokers, with an accompanying reduction in testosterone and an increase in the estradiol concentration in the blood.^11,41^ Genetic alterations in spermatozoa have also been described, since cigarettes contain more than 30 known mutagenic or carcinogenic chemical agents.^41,42^ However, the effects of cigarettes on common sperm parameters (concentration, motility and morphology) are still the subject of discussion and no study has yet been able to demonstrate clear evidence of a dose-response association.^11^ It is important to bear in mind that, in the majority of cases, smoking is just one aspect of a lifestyle that may include the consumption of alcohol, illegal medications or drugs, stress, dietary modifications and other factors that may have synergic or independent effects on sperm quality.

The multiplicity of variables leads to methodological problems that hinder the clear establishment of what impact smoking has on the fertility of the male population as a whole. In the present series, no statistically significant differences in the sperm quality were observed between smoking and non-smoking patients, or between the consumption groups in term of cigarettes/day. One possible explanation for these results is that we only studied fertile individuals, and cases of infertility associated with smoking were excluded from the sample population. Another important matter is the sample size. The group that consumed more than 20 cigarettes/day consisted of only 30 patients. Thus, further studies involving both fertile and infertile men with a large numbers of cases are recommended.

### Caffeine intake

Studies using animal models have suggested that caffeine, when added to the sperm, may increase the motility of the spermatozoa.^43,44^ However, in humans, the results are still inconclusive, mainly because of the difficulty of quantifying the daily consumption of caffeine, in view of the fact that various foodstuffs contain this substance (chocolate and bottled soft drinks, for example). Another important question is the stratification or control of other variables that may increase or decrease sperm motility and thus make it difficult to establish precisely the way in which caffeine affects sperm characteristics.^13^ In our sample, after univariate statistical analysis, coffee consumption remained associated with increased sperm motility.

## CONCLUSIONS

Our sample had a very low percentage of normal sperm morphology, when judged by the WHO standards. Semen volume decreased with age but increased according to the length of sexual abstinence. Sperm concentration was lower in the summer. Sperm motility increased with coffee consumption, but decreased with age, sexual abstinence of greater than or equal to five days, and in the summer months. The normal sperm morphology percentage was lower in summer. Smoking had no influence on the parameters studied.
